# Laser Versus Cold Steel Dissection Tonsillectomy in Recurrent Acute Tonsillitis: A Systematic Review and Meta‐Analysis

**DOI:** 10.1002/lsm.70058

**Published:** 2025-08-25

**Authors:** Marcela Mafra, Thamiris Dias Delfino Cabral, Maria Meritxell Roca Mora, Cynthia Florencio de Mesquita, David Pertzborn, Anna Mühlig, Ferdinand von Eggeling, Orlando Guntinas‐Lichius

**Affiliations:** ^1^ Department of Otorhinolaryngology Jena University Hospital Jena Thuringia Germany; ^2^ Federal Hospital of Bonsucesso Rio de Janeiro Rio de Janeiro State Brazil; ^3^ Medical Faculty International University of Catalonia Barcelona Catalonia Spain; ^4^ Medical Faculty Federal University of Pernambuco Recife Pernambuco Brazil

**Keywords:** CO_2_, diode laser, laser, recurrent acute tonsillitis, surgery, tonsillectomy

## Abstract

**Objectives:**

Severe recurrent acute tonsillitis (RAT) is commonly treated by removing the palatine tonsils, namely tonsillectomy (TE). Laser TE has been suggested as an alternative to cold steel dissection, although its efficacy and safety for the surgical treatment of RAT remain unclear. Therefore, we conducted a systematic review and meta‐analysis comparing laser TE to cold steel dissection TE in patients with RAT.

**Methods:**

We systematically searched PubMed, Embase, and Cochrane Central for eligible studies. Outcomes of interest were intraoperative blood loss, operation time, quality of life, postoperative pain, and postoperative hemorrhage. Subgroup and sensitivity analyses were performed. RStudio v4.1.0 and Review Manager v5.4 were used for statistical analysis. A prospective protocol was registered in PROSPERO (CRD42024533742).

**Results:**

Nine studies, including six randomized trials, met eligibility criteria, comprising 612 patients with RAT. In total, 1224 tonsils were excised; 612 (50%) were submitted to laser TE, and the remaining to cold steel dissection. Laser was associated with lower intraoperative blood loss (mean difference [MD] −35.89; 95% confidence interval [CI] [−53.08, −18.71]; *p* < 0.01; *I*² = 100%) and operation time (MD −10.46; 95% CI [−16.63, −4.29]; *p* < 0.01; *I²* = 99%). No significant differences between interventions were found in postoperative pain, quality of life, or postoperative hemorrhage.

**Conclusions:**

In patients with RAT, laser TE yielded reduced intraoperative blood loss and operation time, with comparable hemorrhage risk, postoperative pain, and quality of life to cold steel dissection. Yet, the occurrence of post‐surgery sore throat remains uncertain, highlighting the need for further randomized trials reporting long‐term outcomes after tonsil surgery.

**Abbreviations**
CI: confidence interval
CO2: carbon dioxide
CONSORT: Consolidated Standards of Reporting Trials
KTP‐532: potassium titanyl phosphate‐532 nanometer
MD: mean difference
NRS: numerical rating scale
PRISMA: Preferred Reporting Items for Systematic Reviews and Meta‐Analysis
PROSPERO: International Prospective Register of Systematic Reviews
RAT: recurrent acute tonsillitis
RCT: randomized controlled trial
ROBINS‐I: Risk of Bias in Non‐Randomized Studies of Interventions
RoB‐2: Risk of Bias Tool for Randomized Trials, version 2
RR: risk ratio
SD: standard deviation
SMD: standardized mean difference
TE: tonsillectomy
VAS: visual analog scale

## 1 Introduction

Recurrent acute tonsillitis (RAT) is characterized by repeated episodes of sore throat due to symptomatic bacterial infection of the palatine tonsils, commonly diagnosed in children and young adults [1]. Given the frequent symptoms, medical visits, and intake of antibiotics, patients with RAT often experience periods of school or work absence, impacting their quality of life, and in a broader perspective, affecting the global economy [2, 3, 4].

The surgical removal of tonsils, namely tonsillectomy (TE), has been a common choice to treat patients with RAT [5, 6]. Recently, the long‐term NATTINA clinical trial supported this trend by reporting that patients who underwent TE presented fewer sore throat episodes in the following years compared to those who received nonsurgical treatment [7]. Since no other method has proven to be superior, total TE performed by cold steel dissection remains the preferred technique in current practice [8]. However, efforts are focused on identifying alternative surgical technologies to achieve the desired outcomes with fewer complications, such as hemorrhage and postoperative pain [9, 10, 11]. Among the alternatives, laser dissection, that is, laser TE, has been investigated as a potential tool [12, 13].

Previous systematic reviews comparing laser to conventional cold steel dissection have been performed and showed promising results [14, 15]. However, they all reported mixed data from patients with different tonsillar conditions, such as RAT, obstructive sleep apnea, and tumors. Therefore, no previous meta‐analysis considering exclusively the subpopulation of patients with RAT has been performed. Furthermore, several studies investigated whether histological findings of chronic inflammation and fibrosis in excised tonsils would be found in patients with a history of recurrent sore throat [16, 17, 18]. Their controversial findings highlighted the uncertainties on whether patients with clinically diagnosed tonsillitis would experience similar outcomes to patients with other diagnoses indicated for TE. Given the pathophysiology of RAT, the demographic diversity among patients with different tonsillar conditions, and the potential benefits of laser dissection over the cold steel technique, we decided to perform a systematic review and meta‐analysis comparing the safety and efficacy outcomes, solely of patients with RAT, who underwent TE performed with laser or cold steel dissection.

## 2 Materials and Methods

This systematic review and meta‐analysis were performed according to the Preferred Reporting Items for Systematic Reviews and Meta‐Analyses (PRISMA) guidelines [19] and the Cochrane Collaboration Handbook for Systematic Reviews of Interventions [20]. The prospective protocol was registered on the International Prospective Register of Systematic Reviews (PROSPERO) in April 2024 (ID: CRD42024533742).

### 2.1 Search Strategy

We systematically searched PubMed, Embase, and Cochrane Central Register of Controlled Trials up to April 10, 2024. The following terms were searched in all three databases: “tonsillectomy,“ or “tonsil resection,” or “tonsil removal,” or “adenotonsillectomy,” or “extracapsular,” and “laser,” or “CO2,” or “carbon dioxide,” or “KTP,” or “potassium titanyl phosphate,” or “thulium,” or “diode,” or “Nd:YAG” and “cold,” or “steel,” or “conventional,” or “traditional,” or “snare.” The complete search strategy is available in the supporting material. Snowballing technique and a complementary search on the website ResearchGate were also performed to identify additional studies [21].

### 2.2 Study Selection

Two authors (M.M. and T.D.D.C.) independently screened articles according to the inclusion and exclusion criteria. Disagreements were resolved via consensus. Studies were included if they met all the following eligibility criteria, based on the PICOS statement: (1) RCTs or non‐randomized cohorts; (2) enrolling patients with the diagnosis of RAT and candidates for total TE, i.e. complete removal of the tonsils; (3) comparing laser dissection TE to (4) conventional cold steel dissection TE; (5) reporting data on at least one of the outcomes of interest. Studies were excluded if they included patients with conditions other than RAT, or if only a partial or intracapsular TE was performed. There was no restriction on follow‐up time or publication year. We excluded studies (1) with no control group, (2) not peer‐reviewed, and (3) not written in English.

### 2.3 Data Collection

Data were extracted from individual studies’ full texts and published appendices for outcomes analyses and baseline characteristics by two authors independently (M.M. and C.F.M.). Three corresponding authors were contacted for additional data, from whom one responded. The number of tonsils instead of the number of patients was considered for data collection and statistical analysis because some included studies used a within‐subject study design, performing intervention and control techniques on the same patient by removing one tonsil with a laser and the contralateral tonsil by cold dissection.

### 2.4 Outcomes of Interest and Subgroup Analyses

The endpoints were (1) intraoperative blood loss; (2) operation time; (3) pain assessment on postoperative day (POD) 1, 3, and 7 (4) quality of life measures, and (5) postoperative hemorrhage. In intraoperative blood loss, outcome measurement was performed using suction bottle and the weight of blood‐soaked absorbent materials, such as gauze and cotton balls. Regarding operation time, some studies considered the period between insertion and removal of the Boyle‐Davis mouth gag, while others relied on the event of the tonsil removal and hemostasis as the time reference. Postoperative hemorrhage was classified as primary or reactionary when occurred within the first 24 h of surgery, and secondary when happened after this period. Primary and secondary hemorrhage were assessed separately. For the outcome of postoperative pain, only data from validated pain scales were considered [22]. Subgroup analysis of RCTs, within‐subject design studies, and laser types were performed to assess the presence of subgroup effects on interventions [23]. Additional sensitivity analyses were performed when applicable, to address heterogeneity among studies.

### 2.5 Quality Assessment

Risk of bias was performed by two investigators independently (M.M. and M.M.R.M.), and disagreements were resolved by consensus. Non‐randomized studies were assessed using the Risk of Bias in Non‐Randomized Studies of Interventions (ROBINS‐I) tool [24], while RCTs were assessed using the Risk of Bias Version 2 (RoB‐2) tool [25].

### 2.6 Statistical Analysis

Binary outcomes were summarized using the Mantel‐Haenszel test, with risk ratios (RR) and 95% confidence intervals (CI) as measures of effect size. Continuous outcomes were compared using mean differences (MD) and standard deviation (SD). When different scales were reported for the same outcome by different studies, standardized mean difference (SMD) was used instead of MD. When median and range were reported in continuous outcomes, we estimated the mean and SD from the median and interquartile ranges [26, 27]. When studies reported standard error instead of SD, the corresponding SDs were manually calculated.

Cochrane *Q* test and *I* ^2^ statistics were used to assess for heterogeneity, where *p* values inferior to 0.10 and *I* ^2^ > 25% were considered significant for heterogeneity. Sources of heterogeneity were investigated using the leave‐one‐out analysis and Baujat plots when *I* ^2^ > 50%. We used random‐effects models for all outcomes. RStudio version 4.1.0 (R Foundation for Statistical Computing, Vienna, Austria) was used with package “meta” for statistical analysis. Review Manager version 5.4 was also used for subgroup analyses (Cochrane Centre, The Cochrane Collaboration, Denmark).

## 3 Results

### 3.1 Study Selection and Characteristics

**Figure 1:**
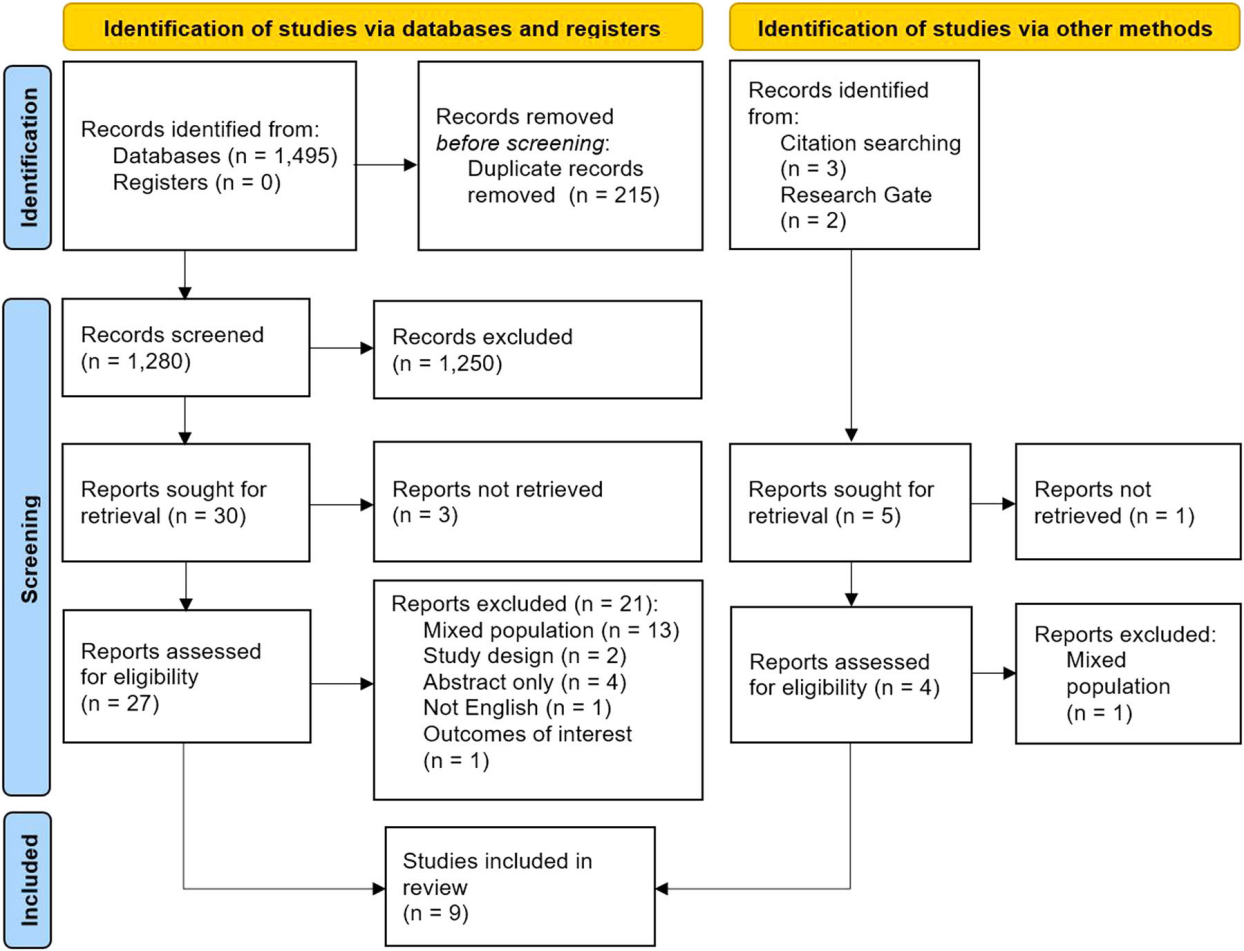
PRISMA 2020 flow diagram.

As detailed in PRISMA flowchart (Figure 1), the initial search on databases yielded 1495 results. After the removal of duplicate records and screening of titles and abstracts, 27 studies were selected for full‐text review, while snowballing and further search resulted in the selection of four additional studies. Thus, 31 studies were fully reviewed based on inclusion and exclusion criteria. A total of nine studies were included in this review, comprising 612 patients from six RCTs [28, 29, 30, 31, 32, 33] and three prospective non‐randomized cohorts [34, 35, 36]. A total of 612 tonsils (50%) were removed with a laser method, and the remaining were resected by cold steel dissection.

Tonsil removal in the intervention arm was performed with CO_2_ laser in four studies [34, 35], diode contact laser in two studies [30, 31], and potassium titanyl phosphate (KTP)‐532 laser in another two studies [33, 36]. All studies included patients with a history of recurrent acute and/or chronic tonsillitis. Three included studies performed a within‐subject study design [29, 35, 36]. Regarding pain scales, the most frequently used was the visual analog scale (VAS), reported by four studies [28, 31, 32, 35]. Definitions of intraoperative blood loss and operation time varied among studies (Tables S1 and S2, respectively). Further baseline characteristics are presented in Table 1.

**Table 1:** Design and characteristics of studies included in the meta‐analysis.

	Study design	Type of laser	Country	Follow‐up (weeks)	Surgical design	Patients, no. laser/control	Tonsils, no. laser/control	Female, no. laser/control	Age, range (years)	Pain assessment scale	Post‐surgery analgesia	Risk of bias assessment
Ali Alharbi, 2015 [28]	RCT	CO_2_	Saudi Arabia	4	Between subject	63/63	126/126	NA	16–45	VAS	Diclofenac and paracetamol	Some concerns^b^
Baghdasaryan, 2024 [29]	RCT	Thulium	Armenia	1.86	Within subject	24/24	24/24	10^a^	17–42	NRS	Analgesics	Some concerns^b^
Elbadawey, 2015 [30]	RCT	Diode	Saudi Arabia	2	Between subject	40/40	80/80	22/21	5–15	W‐B FACES	Analgesics	Low risk^b^
Gandhi, 2019 [34]	Prospective non‐randomized	CO_2_	India	NA	Between subject	20/20	40/40	11/12	5–35	NA	NA	Some concerns^d^
Ghiyali, 2023 [35]	Prospective non‐randomized	CO_2_	India	6	Within subject	25/25	25/25	11^a^	NA^c^	VAS	Paracetamol	Some concerns^d^
Ifthikhar, 2018 [31]	RCT	Diode	Pakistan	24	Between subject	50/50	100/100	21/18	5–15	VAS	NA	Low risk^b^
Oas, 1990 [36]	Prospective non‐randomized	KTP‐532	USA	2	Within subject	31/31	31/31	NA	10–35	Pain localization to side left/right	Paracetamol and codeine	Some concerns^d^
Thangavel, 2018 [32]	RCT	CO_2_	India	1	Between subject	63/63	126/126	37/33	7–18	VAS	Ibuprofen and paracetamol	Low risk^b^
Vijayasundaram, 2019 [33]	RCT	KTP‐532	India	4	Between subject	30/30	60/60	12/18	4–18	W‐B FACES and NRS	Diclofenac	Low risk^b^

Abbreviations: CO_2_, carbon dioxide; KTP, potassium titanyl phosphate; NA, not available information; NRS, numerical rating scale; RCT, randomized controlled trial; VAS, visual analog scale; W‐B FACES, Wong‐baker faces pain scale.

^a^ Only the total number of female patients was available

^b^ Assessment performed with risk of bias tool, version 2 (ROB‐2).

^c^ Age was reported in mean ± standard deviation: 12.32 ± 8.27 years.

^d^ Assessment performed with risk of bias in non‐randomized studies—of interventions (ROBINS‐I).

### 3.2 Pooled Analyses of Studies

**Figure 2:**
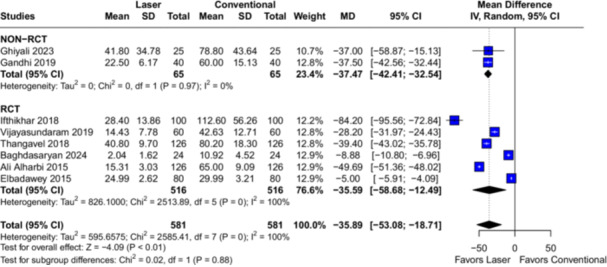
Comparison of laser and conventional (cold steel) dissection for the outcome of intraoperative blood loss. On the left side of the figure, each horizontal line represents an individual included study. On the right side of the figure, the dashed vertical line represents the meta‐analysis overall measure of effect. Intraoperative blood loss was significantly lower in the laser dissection group in the overall analysis. CI, confidence interval; MD, mean difference; RCT, randomized controlled trial; SD, standard deviation.

**Figure 3:**
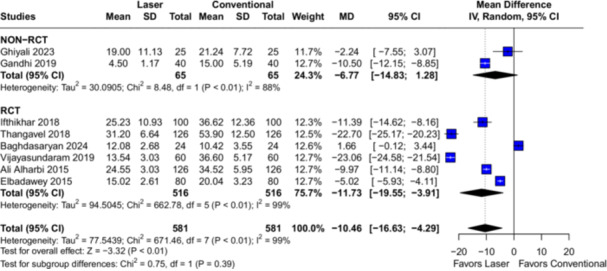
Comparison of laser and conventional (cold steel) dissection for the outcome of operation time. On the left side of the figure, each horizontal line represents an individual included study. On the right side of the figure, the dashed vertical line represents the meta‐analysis overall measure of effect. Operation time was significantly shorter in the laser dissection group in the overall analysis. CI, confidence interval; MD, mean difference; RCT, randomized controlled trial; SD, standard deviation.

In the overall analysis with eight studies (*n* = 1162 tonsils), the use of laser was associated with reduced intraoperative blood loss (MD −35.89; 95% CI [−53.08, −18.71]; *p* < 0.01; *I*² = 100%; Figure 2) and shorter operation time (MD −10.46; 95% CI [−16.63, −4.29]; *p* < 0.01; *I*² = 99%; Figure 3) when compared to cold steel dissection.

**Figure 4:**
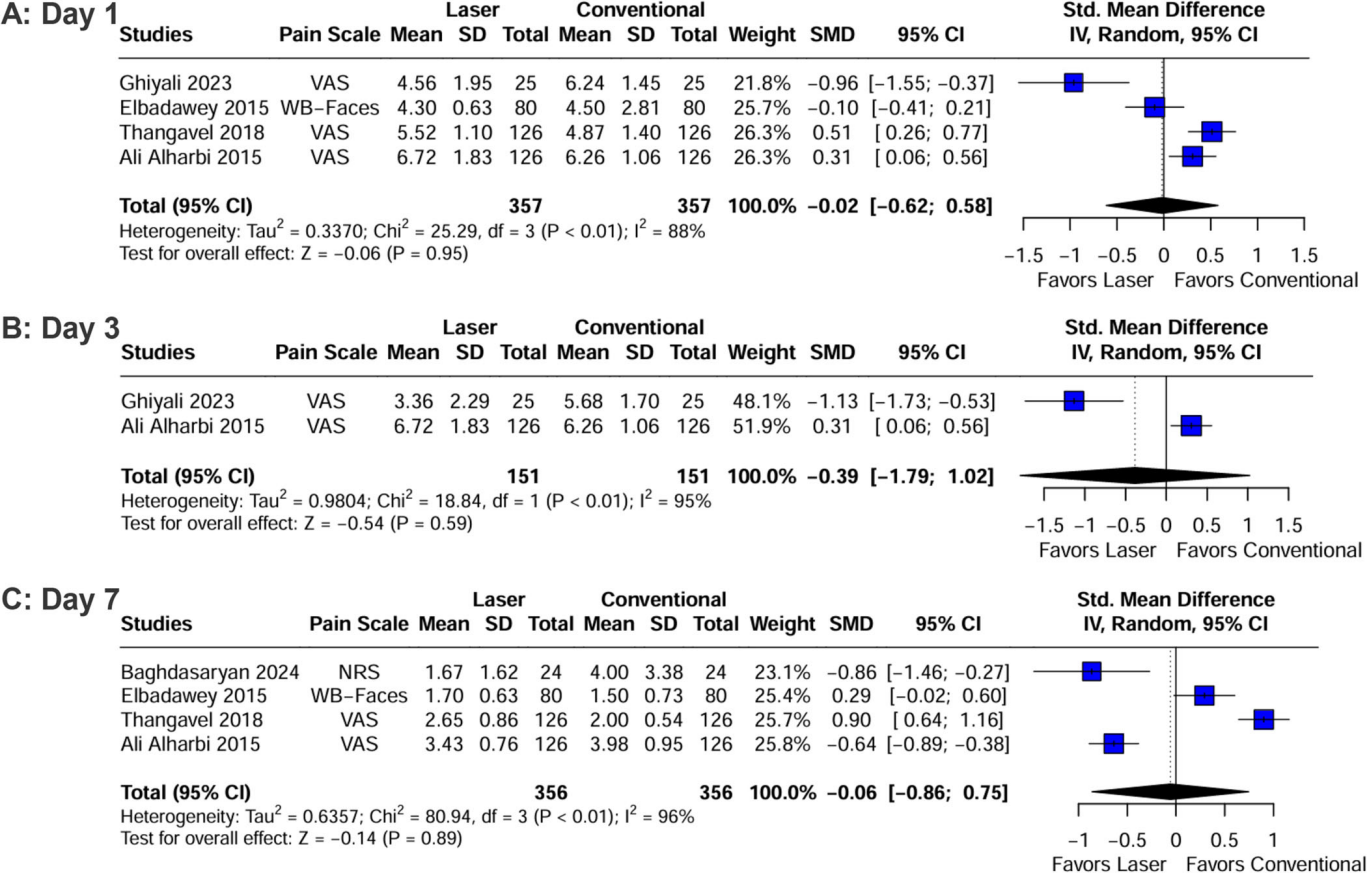
Comparison of laser and conventional (cold steel) dissection for the outcome of postoperative pain on different postoperative days (PODs). A: POD 1; B: POD 3; C: POD 7. On the left side, each horizontal line represents an individual included study. On the right side, the dashed vertical line represents the meta‐analysis overall measure of effect. Postoperative pain was not statistically different between laser and cold steel dissection, regardless of the POD. CI, confidence interval; NRS, numerical rating scale; SD, standard deviation; SMD, standardized mean difference; VAS, visual analog scale; WB‐Faces, Wong‐Baker Faces.

In postoperative pain assessment, no difference in effect size was observed between patients operated with laser or cold steel dissection, regardless of the postoperative day. On POD 1, pooling data of four studies (*n* = 714 tonsils) yielded no difference in effect size between groups (SMD −0.02; 95% CI [−0.62, 0.58]; *p* = 0.95; *I*² = 88%; Figure 4A). On POD 3, two studies (*n* = 302 tonsils) reported data that resulted in no difference in effect size between laser and cold steel dissection groups (SMD −0.39; 95% CI [−1.79, 1.02]; *p* = 0.59; *I*² = 95%; Figure 4B). On POD 7, the analysis of four studies (*n* = 712 tonsils) also showed no difference in effect size between groups (SMD −0.06; 95% CI [−0.86, 0.75]; *p* = 0.89; *I*² = 96%; Figure 4C).

As quality of life outcome measures, time to return to daily activities and to normal diet were reported by one study, which yielded no statistical difference between intervention groups [30]. Another study described an earlier return to activities and swallowing function in patients from the laser dissection group; however, no quantitative data were reported [34]. Furthermore, three studies observed patients’ tonsillar fossae healing status, by subjective evaluation of the medical team in the follow‐up visits after surgery [29, 33, 36].

**Figure 5:**
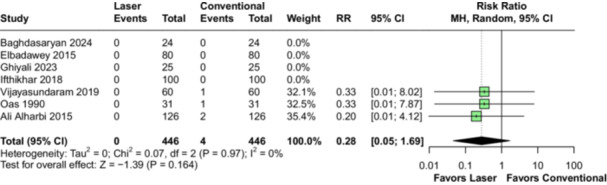
Comparison of laser and conventional (cold steel) dissection for the outcome of primary hemorrhage. On the left side of the figure, each horizontal line represents an individual included study. On the right side of the figure, the dashed vertical line represents the meta‐analysis overall measure of effect. Primary hemorrhage was not statistically different between laser and cold steel dissection. CI, confidence interval; RR, risk ratio.

**Figure 6:**
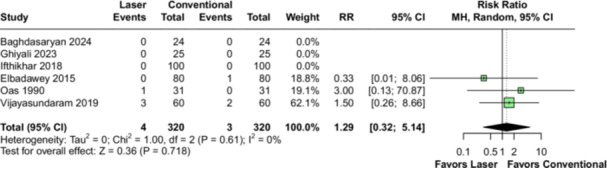
Comparison of laser and conventional (cold steel) dissection for the outcome of secondary hemorrhage. On the left side of the figure, each horizontal line represents an individual included study. On the right side of the figure, the dashed vertical line represents the meta‐analysis overall measure of effect. Secondary hemorrhage was not statistically different between laser and cold steel dissection. CI, confidence interval; RR, risk ratio.

The incidence of bleeding complications was reported by seven studies (*n* = 892 tonsils). There were no significant differences in the occurrence of primary hemorrhage (RR 0.28; 95% CI [0.05, 1.69]; *p* = 0.164; *I*² = 0%; Figure 5) or secondary hemorrhage (RR 1.29; 95% CI [0.32, 5.14]; *p* = 0.718; *I*² = 0%; Figure 6) between patients undergoing laser or cold steel dissection TE.

### 3.3 Subgroup and Sensitivity Analyses

The pre‐specified assessment of RCTs and non‐randomized studies in the subgroup analysis for the outcomes of intraoperative blood loss (Figure 2) and operation time (Figure 3) yielded no differences between subgroups (*p* = 0.88 and *p* = 0.39, respectively). These findings suggest that the subgroups are comparable, indicating that randomization might not be an influencing factor to the significant heterogeneity observed in the pooled analyses.

An additional subgroup analysis to clarify whether the within‐subject design could have influenced the pooled analyses was performed for the outcomes of intraoperative blood loss and operation time [37]. For the outcome of operation time, a significant difference between subgroups (*p* < 0.0001, Supporting Information S1: Figure S1) highlighted the different time frames considered in each subgroup. In the within‐subject studies, operation time was defined as the time to remove one tonsil and achieve unilateral hemostasis, while in the between‐subject studies, the operation time included bilateral tonsillar removal. Given that the within‐subject subgroup comprised approximately 10% of the overall population and 24% of overall weight for this outcome, the pooled studies result seems to reflect the findings obtained from the between‐subject design subgroup. Similarly, for the outcome of intraoperative blood loss, the overall results also seem to reflect the results obtained from the between‐subject design subgroup, despite no difference was found in the subgroup analysis (*p* = 0.27, Supporting Information S1: Figure S2).

The subgroup analysis of laser types showed a statistically significant difference among laser subgroups for the outcomes of intraoperative blood loss and operation time (*p* < 0.00001 in both cases, Supporting Information S1: Figures S3 and S4). Despite that, the results in the subgroups of CO_2_ and diode, which had more than two studies, remained consistent with the pooled analysis of eight studies. One exception was for the outcome of intraoperative blood loss in the cohort of diode laser studies, where no statistical difference was found between intervention groups, which could be attributed to the lower statistical power given by only two studies and a limited population, when compared to the overall analysis.

Through leave‐one‐out analysis and Baujat plots, we evaluated the contribution of individual studies to heterogeneity. In operation time, no study seemed to predominantly influence heterogeneity. Although one study was spotted as highly influencing results and heterogeneity (Supporting Information S1: Figure S5), omitting this study did not reduce heterogeneity (Supporting Information S1: Figure S6) [33]. Similarly, removing any of the included studies did not change heterogeneity, and results after sensitivity analysis remained consistent with the pooled analysis (Supporting Information S1: Figure S6). In intraoperative blood loss, two studies could be pinpointed as outliers. One study contributed to the overall heterogeneity [28], while the other study influenced the overall results (Supporting Information S1: Figure S7) [30]. Despite that, the leave‐one‐out analysis showed that none of the studies removed substantially changed heterogeneity and the results after sensitivity analysis remained in line with the full analysis (Supporting Information S1: Figure S8). Thus, in both operation time and intraoperative blood loss analyses, intrinsic differences among all studies may have a major role in the overall heterogeneity, mainly arising from the variability in the methods used to measure the outcomes.

To address this hypothesis, we performed a post‐hoc subgroup analysis (Supporting Information S1: Figure S9), relying on similar definitions of outcome measurements (Supporting Information S1: Table S1). Among the studies reporting operation time measures, we were able to combine studies in three subgroups, each with two studies, using the following criteria: (1) within‐subject studies which considered the time between first incision and complete hemostasis for each tonsil; (2) operation time between first incision and complete hemostasis of both tonsils; (3) operation time from insertion to removal of Boyle‐Davis mouth gag. Two studies from our pooled analysis did not fit in these criteria. The results of this analysis yielded a statistically significant subgroup effect (*p* < 0.0001), showing that these subgroups are different from each other, and the heterogeneity within each subgroup was significantly lower than in the pooled analysis (*I* ^2^ = 0%–46%). These findings corroborate our hypothesis that the main source of heterogeneity in the pooled analysis are given by the variability in the outcome measurements.

For the outcome of postoperative pain, sensitivity analysis was performed for POD 1 and POD 7. On POD 1, no study dominance could be demonstrated from the Baujat plot (Supporting Information S1: Figure S10), while on POD 7, two studies were highly influencing results and heterogeneity (Supporting Information S1: Figure S11) [28, 32]. The leave‐one‐out analysis showed that none of the studies substantially decreased heterogeneity when removed (Supporting Information S1: Figures S12 and S13), which suggests the presence of variable factors across several studies. Given the subjective nature of pain perception reported by patients, this is likely to be a crucial factor in the outcome assessment. Additionally, the analgesic regimen varied among included studies (Table 1), which may also have contributed to the variability of pain scores reported, consequently reflecting the overall heterogeneity. Despite that, results in sensitivity analysis remained consistent with the full analysis, with no statistical significance between intervention groups for this outcome.

### 3.4 Risk of Bias Assessment

In quality appraisal of individual RCTs, two studies were evaluated with “some concerns” because of the methods used to measure the outcomes of intraoperative blood loss and pain, respectively (Supporting Information S1: Figure S14) [28, 29]. The remaining studies were considered to have “low” risk of bias. One included study did not follow the Consolidated Standards of Reporting Trials (CONSORT) statement guidelines for randomized trials [38], therefore it was considered as a non‐randomized study [36]. In the assessment of non‐randomized cohorts, all three studies were found to have “moderate” risk of bias due to minimal confounding bias (Supporting Information S1: Figure S15) [34, 35, 36]. The funnel plot presented asymmetry by visual inspection (Supporting Information S1: Figure S16), which suggests potential evidence of small‐study effect that may be attributable to publication bias. Egger's regression test to investigate publication bias was not performed due to the limited amount of studies [39].

## 4 Discussion

In this systematic review and meta‐analysis of nine studies, comprising 612 patients with RAT, we investigated the efficacy and safety of laser compared to conventional cold steel dissection in total TE. Our main findings were (1) lower intraoperative blood loss, (2) shorter operation time, and (3) comparable postoperative pain and hemorrhage risk using laser, when compared to cold steel technique.

Our overall results are consistent with a previous meta‐analysis that included patients with different clinical diagnoses who underwent TE and compared CO_2_ laser to cold steel dissection [15]. The remarkable effects of laser on live tissue include coagulation and evaporation, resulting in the desired outcomes of cutting and hemostasis during surgical resection [40]. In these circumstances, the efficacy in achieving hemostasis directly relates to the amount of intraoperative blood loss, which consequently determines the time needed to complete the surgery, namely the operation time. When using cold steel dissection, hemostasis is achieved by ligature, local pressure, and/or using other hot methods [28, 30, 31]. Therefore, our findings of lower intraoperative blood loss and operation time when using laser dissection versus cold steel dissection align with the anticipated scenario.

Yet, quantitative comparisons of these results should be made with reservations. Due to the quantitative nature of the outcome measurements and the absence of a standardized method to measure these outcomes, the pooled analysis of studies is naturally prone to interstudy variability. However, the qualitative comparison between intervention groups remains consistent, since each study follows their defined outcome measurement criteria in both intervention groups. Therefore, our findings reflect a reliable qualitative analysis between laser and cold steel dissection and may highlight the need for outcome measures standardization in future studies.

The inclusion of different laser types in our analyses does not seem to strongly influence these results. A previous review investigating the performance of various lasers, such as CO_2_, KTP‐532, and diode laser in endonasal surgery, suggested that all laser types can provide favorable clinical outcomes, even though their physical properties can vary considerably. For instance, CO_2_‐laser properties enable high energy absorption by water molecules in the tissue. Consequently, deep tissue damage is hampered, and two clinically relevant applications of this laser become possible: a focused high‐powered density use that leads to vaporization and tissue ablation, but also an unfocused mode that leads to coagulation [41]. On the other hand, diode lasers enable milder tissue absorption, which allows for increased scattered radiation within the tissue, leading to deeper tissue penetration and lower ablative properties. However, by applying the diode laser in direct contact with the tissue, ablation and coagulation can be enhanced. The contact to the tissue provides a higher power density that promotes ablation, changing the tissue local properties, which increases energy absorption, decreases scattering and promotes improved clinical outcome to this type of laser [40]. Therefore, it is possible to hypothesize that, despite the diversity among laser properties, they can perform well in surgical applications when the appropriate power settings are used and the surgeon is able to effectively apply the laser beam to the tissue [40]. This perspective would corroborate our findings that demonstrated an existing subgroup effect for CO and diode laser, which suggests that the laser types may influence the effects of interventions. However, for the outcome of operation time, the results in both subgroups individually favored laser dissection, in the same manner as the overall analysis. For the outcome of intraoperative blood loss, the diode laser subgroup demonstrated no difference between interventions, which could be related to its ablative properties. Furthermore, the remaining heterogeneity within each subgroup suggests that the variety of definitions of outcome measures previously discussed is also a factor in this circumstances. As a result, the observed qualitative subgroup effect could be attributed to intrinsic features of the different types of laser, yet promoting favorable clinical outcomes for patients. In addition, a study comparing Ho:YAG laser to conventional TE (n = 10 patients) reported similar results in operation time and intraoperative bleeding. Interestingly, the same study also compared conventional TE to the Er,Cr:YSGG laser (*n* = 10 patients), initially designed for dental procedures, which yielded increased operation time and comparable intraoperative blood loss, possibly due to its suboptimal application in palatine tonsil surgery [42]. In light of the laser properties and our findings, the conditions observed in endonasal surgery, mainly nasal turbinates surgery [40], may also apply to tonsil surgery, and no definitive laser technology is superior to others.

Cost‐effectiveness is an additional aspect of using lasers in surgery that deserves attention. In tonsil surgery, a recent trial has reported lower overall costs and a probability of cost‐effectiveness of 71% when using CO2‐laser for partial TE, compared with cold dissection total TE [43]. Furthermore, an audit‐based investigation performed in the United Kingdom found at least a comparable cost‐effectiveness of using coblation versus cold steel dissection TE in children and adults [44]. Yet, data on the cost‐effectiveness of laser TE are still scarce, and none of the included studies in this review reported such data.

Among the postoperative complications, the occurrence of hemorrhage is infrequent, yet relevant due to the potential life‐threatening risk and need for invasive actions [11, 45]. The comparable primary and secondary hemorrhage risks between intervention groups found in our analysis are in line with previous reports, but controversy still exists. A retrospective study using coblation versus cold dissection for TE [46], and a review comparing coblation to other methods, including cold dissection [47], reported results similar to ours. On the other hand, a large prospective multicenter non‐randomized study reported that patients after cold dissection TE had fewer hemorrhage complications when compared to patients operated with a hot technique such as coblation and bipolar diathermy [48]. Furthermore, a review of RCTs comparing coblation to laser dissection reported no statistical difference between interventions in postoperative hemorrhage [49]. Given the current evidence and the remaining uncertainties, further high‐quality evidence seems necessary to draw more accurate conclusions about the performance of laser versus cold dissection in the incidence of bleeding complications in patients with RAT undergoing TE.

In addition to that, the occurrence of postsurgical hemorrhagic events may be particularly investigated when patients undergo a within‐subject study design with different interventions. In this setting, the results reflect the performance of the interventions used on each surgical site, but also the ability of the surgeon to operate on the right or the left side of the patient. A recent report has demonstrated a difference in postsurgery hemorrhage after bilateral tonsillectomy performed by electrocautery, with a higher risk of bleeding on the side operated by the surgeon's nondominant hand [50]. Considering that, attention should be given when different interventions are compared on each side of the same patient. In our pooled analysis, both studies using a within‐subject design reported no hemorrhagic events on either intervention groups, which statistically produce no contribution to the overall results. Yet, clinical trials that choose to use this experimental design should account for this potential source of bias when reporting their results.

The postoperative pain after TE is known to be very intense, even compared to major abdominal and thoracic surgeries [51]. For that reason, investigating postoperative pain is essential to determine whether patients report similar levels of pain with different dissection techniques. In a study comparing Er,Cr:YSGG and Ho:YAG lasers to conventional TE (*n* = 10 patients in each comparison), patients reported higher pain scores in the laser group [42]. On the other hand, our results are consistent with a previous review, demonstrating no significant difference in pain perception between intervention groups [15]. Yet, according to the literature, variations in pain scale performance when assessing pain can be influenced by the medical care team's competence in applying the scale and the patient's understanding of the scale system [52, 53, 54]. Furthermore, our post hoc sensitivity analysis of within‐subject studies aimed at evaluating whether this methodological aspect would influence the perception of subjective outcomes such as pain. Considering that the same patient is treated with both interventions, a possible true difference in the perception of pain reported by the patient could be highly influenced by the contralateral healing site, impacting the outcome assessment. Our findings showed that this methodological aspect did not solely contribute to heterogeneity or influence the overall results. In this scenario, other factors such as individual pain perception, which is an intrinsic attribute of assessing pain in any circumstance, and the diversity of analgesia management among studies may influence the postoperative pain reported. In addition, intracapsular partial TE, known as tonsillotomy, is an established technique to treat children with obstructive sleep apnea [55]. The morbidity, especially postoperative bleeding and pain, is much less after partial tonsil resection compared to standard total TE [56]. An ongoing RCT is analyzing if laser and other tonsillotomy techniques are as effective as TE also for patients with RAT [57].

Besides postoperative pain, clinical trials frequently investigate quality‐of‐life outcomes such as return to daily activities and return to normal diet when comparing different TE dissection technologies [58, 59, 60, 61]. Despite that, only two out of the nine included studies in this review reported those outcomes [30, 34]. Furthermore, the subjective evaluation of healing status reported by three studies restricted a quantitative analysis of this data [29, 33, 36]. Therefore, this scenario highlights the importance of future studies focusing more on these outcomes, as they provide valuable information about the overall patients’ experience after undergoing this surgery.

The decision to operate on patients with RAT primarily aims to enhance their overall quality of life by reducing the negative impact of recurring sore throat episodes [5, 7]. Previous studies showed an improvement in this aspect at least 6 months after TE, in patients with a history of recurrent tonsillitis, compared to baseline [2, 62]. Another study evaluated the quality of life of patients compared to a healthy population [63]. More recently, an RCT reported that post‐baseline patient satisfaction was comparable between laser tonsillotomy and cold steel TE. Still, patients who underwent tonsillotomy with laser reported more persistent symptoms at both 1 and 2 years postprocedure compared to those treated with total TE with cold steel [43]. In this context, residual tonsillar tissue at the surgical site may contribute to these persistent symptoms and increase the likelihood of requiring a secondary procedure [64]. Therefore, the performance of the laser in total TE may not be followed by the same persistent symptoms. These factors should be taken into consideration when surgeons are deciding between total or partial dissection, performed with laser or cold steel.

However, studies comparing different dissection techniques do not often report long‐term quality‐of‐life outcomes such as sore throat episodes after tonsil removal [28, 29, 30, 31, 32, 33, 34, 35, 36]. One reason for that may be the additional resources necessary to keep long‐term follow‐up of patients after TE. Nevertheless, documenting episodes of sore throat after surgery and overall quality of life as outcome measures after TE may give valuable information for future guideline recommendations and public health and economic strategies [7, 65].

In light of the current practice, cold steel dissection remains the conventional choice for total TE, as no other method succeeded in overcoming it. Although the use of lasers in TE has long been investigated, the benefits of this approach, particularly in patients with RAT, are still unclear. Our findings elucidate that the performance of laser dissection compared to cold steel dissection in patients with RAT undergoing TE may be similar to the general population undergoing TE. Accordingly, the laser technique provides a faster and more hemodynamically stable dissection during tonsil removal. Despite that, the impact of laser dissection on postoperative sore throat episodes and overall quality of life in patients with a history of RAT is still unknown. Given this scenario, it is essential to conduct high‐quality randomized trials with extended follow‐up periods to evaluate long‐term performance of laser dissection.

This study has important limitations. The different definitions used to measure intraoperative blood loss and operation time in individual studies may have introduced a certain amount of variability among studies, for which we performed sensitivity analyses to assess sources of heterogeneity. Quantitative interpretations of the reported data should be done with reservations. Moreover, some studies used a within‐subject surgical design, while others performed a pairwise between‐subject design. Particularly in pain assessment, these different designs could have played an important role, for which sensitivity analysis was performed. Furthermore, the sensitivity analyses performed to investigate the influence of certain laser types on the results were performed only for certain lasers, according to data availability. Additional clinically relevant outcomes of quality of life and recurrence of sore throat, as well as cost‐effectiveness, were not reported by the included studies, which restricted further analyses.

## 5 Conclusion

This meta‐analysis compared the use of laser dissection to cold steel dissection in patients with RAT undergoing TE. Laser dissection was found to be associated with lower intraoperative blood loss and operation time, yet no differences were observed in postoperative pain and hemorrhage complications between the intervention groups. Our findings suggest that patients with RAT benefit from laser dissection similarly to the general population who undergo TE. These results underscore the need for future studies to generate long‐term, clinically meaningful data and to further evaluate the risk of secondary procedures and persistent postoperative symptoms in this specific population.

## Disclosure

All authors take responsibility for all aspects of the reliability and freedom from bias of the data presented and their discussed interpretation.

## Ethics Statement

The authors have nothing to report.

## Consent

The authors have nothing to report. The methodological procedures of this study followed the PRISMA statement.

## Conflicts of Interest

The authors declare no conflicts of interest.

## Supporting information

Supplementary_material_revision_v2.
