# Supplementary material for: Laser Versus Cold Steel Dissection Tonsillectomy in Recurrent Acute Tonsillitis: A Systematic Review and Meta‐Analysis
Source: Lasers Surg Med. 2025 Aug 25;57(8):645–57. doi: 10.1002/lsm.70058 (PMC12411692; doi:10.1002/lsm.70058)
Supplement: Supplementary file 1 — Supplementary_material_revision_v2. [file LSM-57-645-s001.docx]

**Recurrent Acute Tonsillitis: Laser Tonsillectomy Versus Cold Steel Tonsillectomy - A Systematic Review and Meta-Analysis**

Marcela Mafra (M.D.),^1^ Thamiris Dias Delfino Cabral (M.D.),^2^ Maria Meritxell Roca Mora (M.D.),^3^ Cynthia Florencio de Mesquita,^4^ David Pertzborn (Ph.D.),^1^ Anna Mühlig (Ph.D.),^1^ Ferdinand von Eggeling (Prof., Ph.D.),^1^ Orlando Guntinas-Lichius (Prof., M.D.)^1^

^1^ Department of Otorhinolaryngology, Jena University Hospital, Jena, Germany

^2^ Federal Hospital of Bonsucesso, Rio de Janeiro, Brazil

^3^ Medical Faculty, International University of Catalonia, Barcelona, Spain

^4^ Medical Faculty, Federal University of Pernambuco, Recife, Brazil

**Supplementary material**

Supplemental methods Page 2

Supplemental figures S1-S16 Page 3

Supplemental tables S1-S2 Page 14

**Supplemental Methods**

**Search strategy used in Pubmed, Embase and Cochrane Central databases**

(tonsillectomy OR tonsillectomies OR tonsilectomy OR extracapsular OR "tonsil resection" OR “tonsils resection” OR "tonsil resections" OR “tonsils resections” OR "tonsil removal" OR "tonsils removal" OR “tonsil surgery” OR “tonsil surgeries” OR adenotonsillectomy OR adenotonsillectomies) AND

(laser OR "carbon dioxide" OR "CO2" OR "potassium titanyl phosphate" OR KTP OR thulium OR diode OR “Nd:YAG” OR “neodymium-doped yttrium aluminum garnet”) AND

(cold OR resection OR dissection OR dissections OR traditional OR standard OR steel OR snare OR conventional)

**Supplemental Figures**


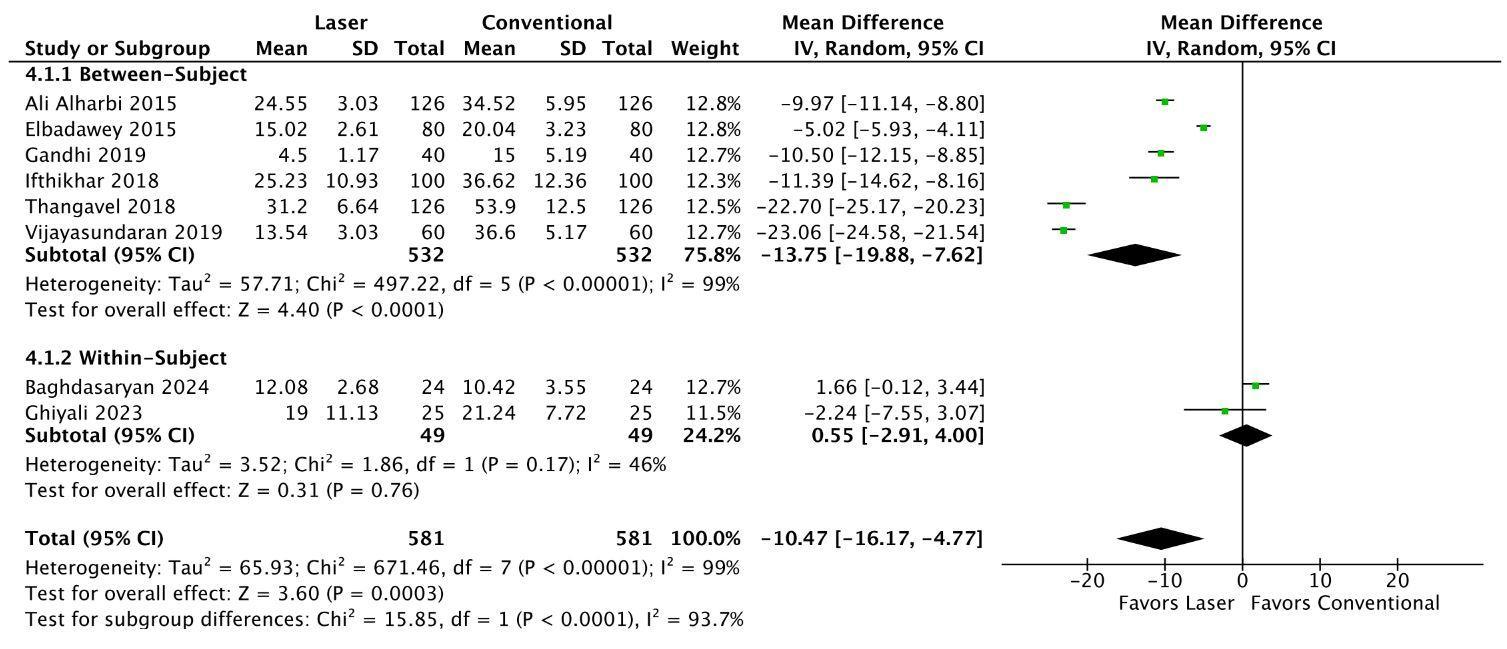


**Figure S1: Subgroup analysis of within-subject design studies for the outcome of operation time.** On the left side of the figure, each horizontal line represents an individual included study. On the right side of the figure, the dashed vertical line represents the meta-analysis overall measure of effect. Operation time was significantly lower in the laser dissection group in the overall analysis. SD: Standard deviation; CI: Confidence interval.


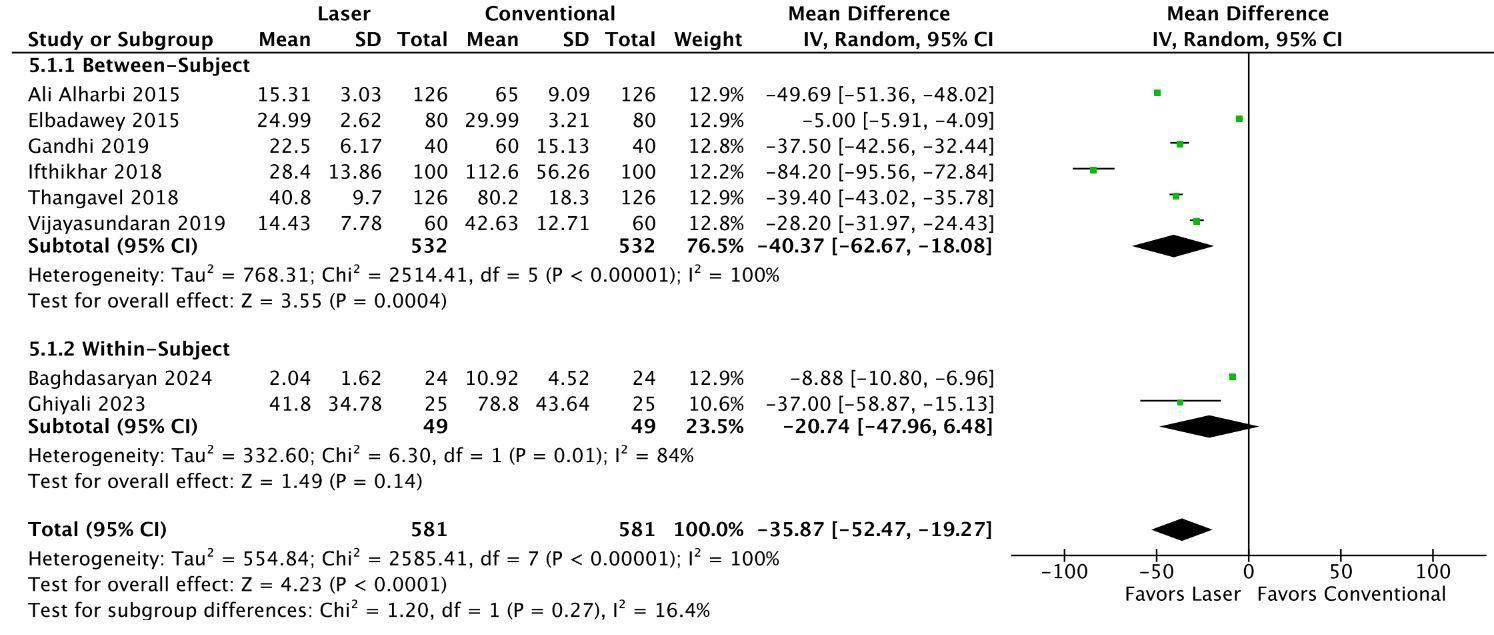


**Figure S2: Subgroup analysis of within-subject design studies for the outcome of intraoperative blood loss.** On the left side of the figure, each horizontal line represents an individual included study. On the right side of the figure, the dashed vertical line represents the meta-analysis overall measure of effect. Intraoperative blood loss was significantly lower in the laser dissection group in the overall analysis. SD: Standard deviation; CI: Confidence interval; IV: Inverse variance.


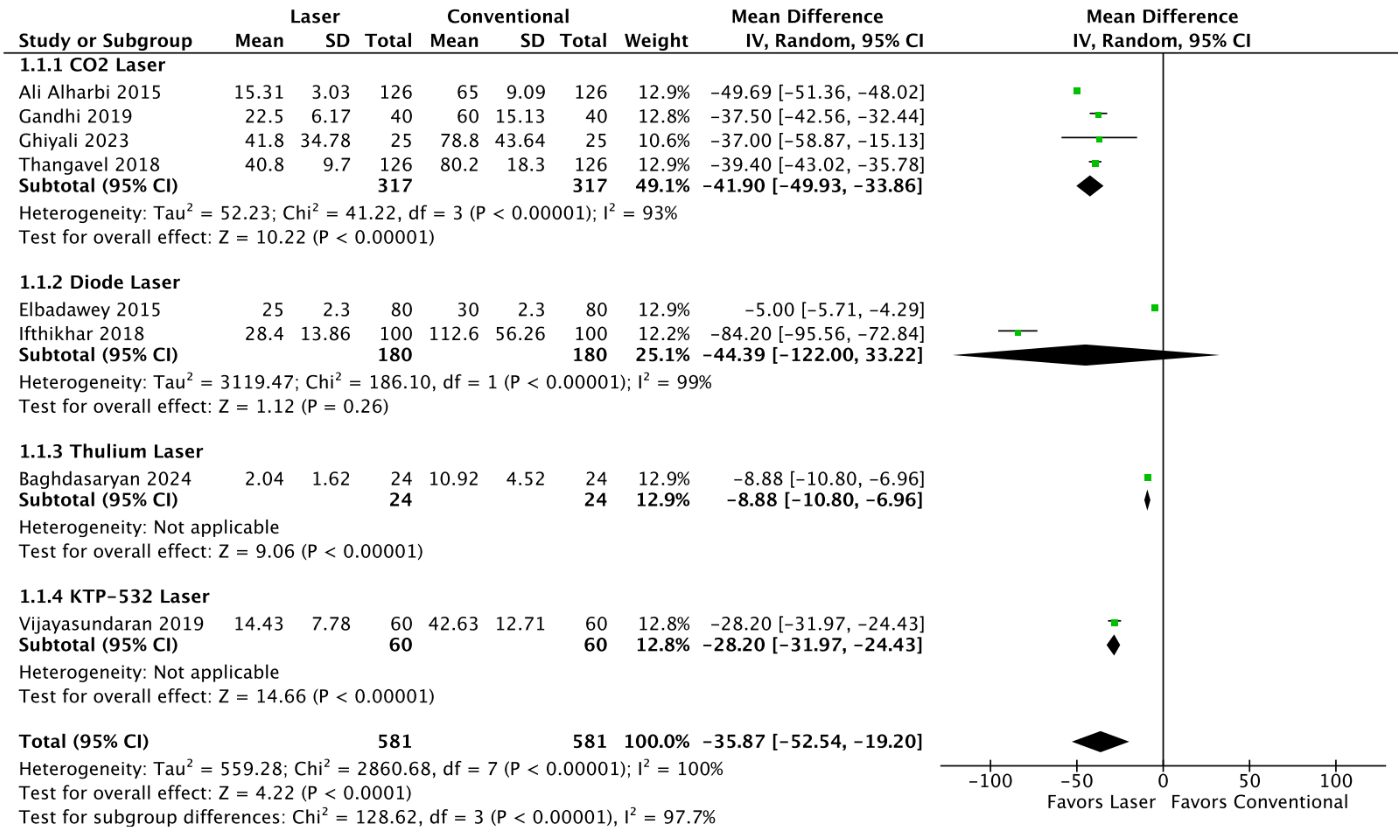


**Figure S3: Subgroup analysis by laser type for the outcome of intraoperative blood loss.** On the left side of the figure, each horizontal line represents an individual included study. On the right side of the figure, the dashed vertical line represents the meta-analysis overall measure of effect. Intraoperative blood loss was significantly lower in the laser dissection group in the overall analysis. CO2: carbon dioxide; KTP: Potassium tytanil phosphate; SD: Standard deviation; CI: Confidence interval; IV: Inverse variance.


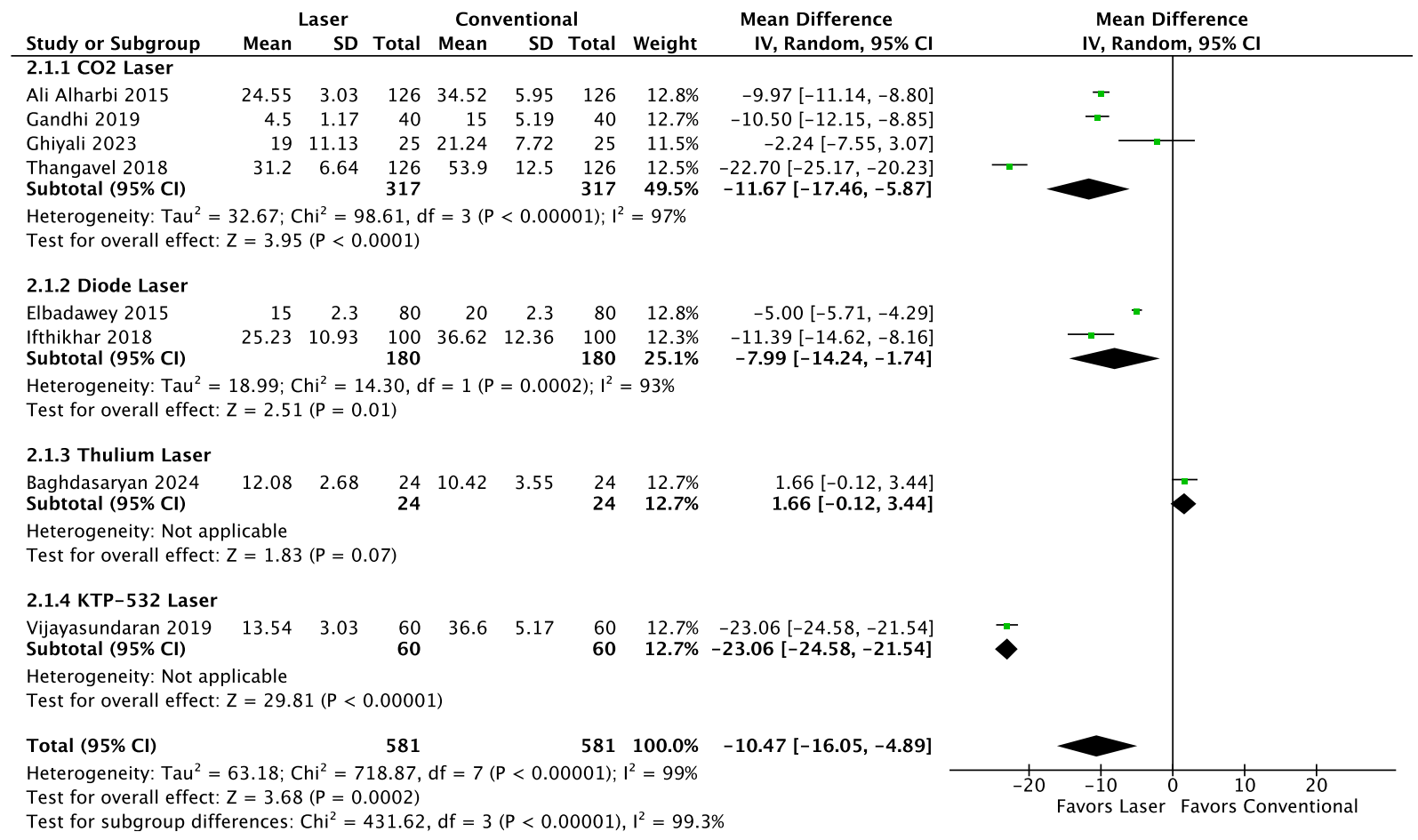


**Figure S4: Subgroup analysis by laser type for the outcome of operation time.** On the left side of the figure, each horizontal line represents an individual included study. On the right side of the figure, the dashed vertical line represents the meta-analysis overall measure of effect. Operation time was significantly lower in the laser dissection group in the overall analysis. CO2: carbon dioxide; KTP: Potassium tytanil phosphate; SD: Standard deviation; CI: Confidence interval; IV: Inverse variance.


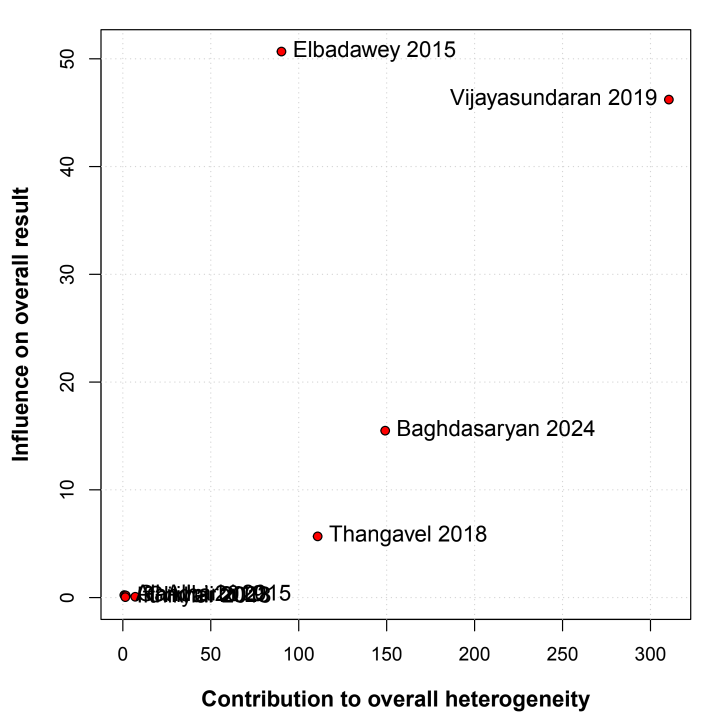


**Figure S5: Baujat plot for the outcome of operation time.** On the x-axis, the contribution of a study to heterogeneity is presented, while on the y-axis, the influence of this study to the overall heterogeneity. The location of each study on the plot represents how impactful this study is to the overall analysis.


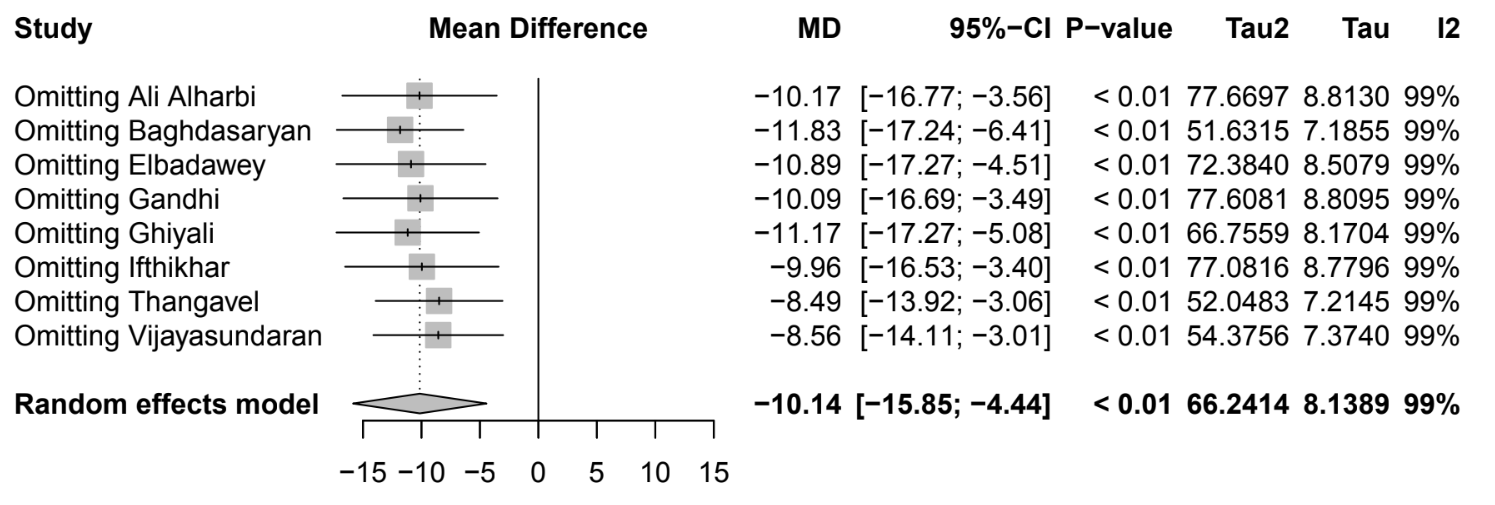


**Figure S6: Leave-one-out analysis for the outcome of operation time.** Each line shows the meta-analysis for this outcome without including the omitted study (according to the first column on the left). Excluding any of the individual studies changed statistical significance or heterogeneity.


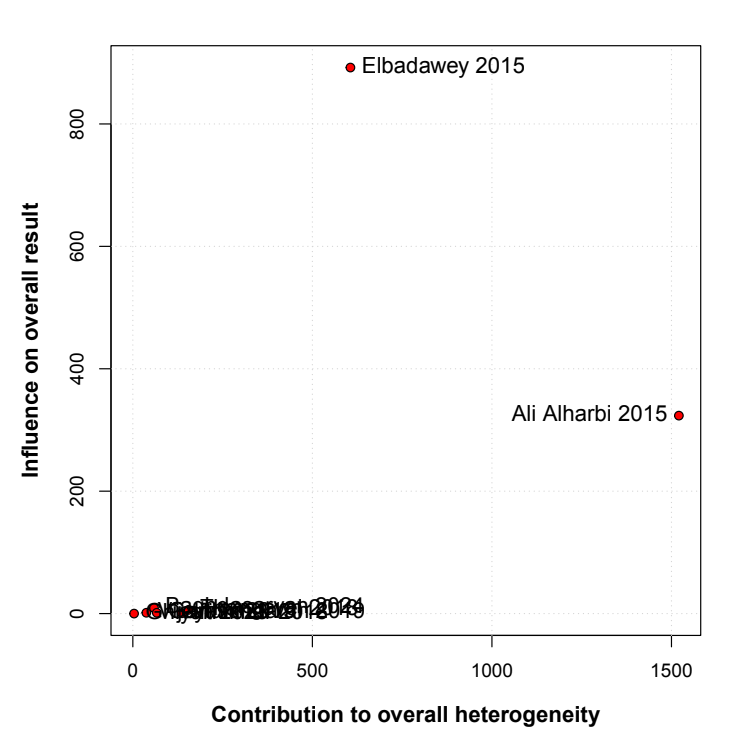


**Figure S7: Baujat plot for the outcome of intraoperative blood loss.** On the x-axis, the contribution of a study to heterogeneity is presented, while on the y-axis, the influence of this study to the overall heterogeneity. The location of each study on the plot represents how impactful this study is to the overall analysis.


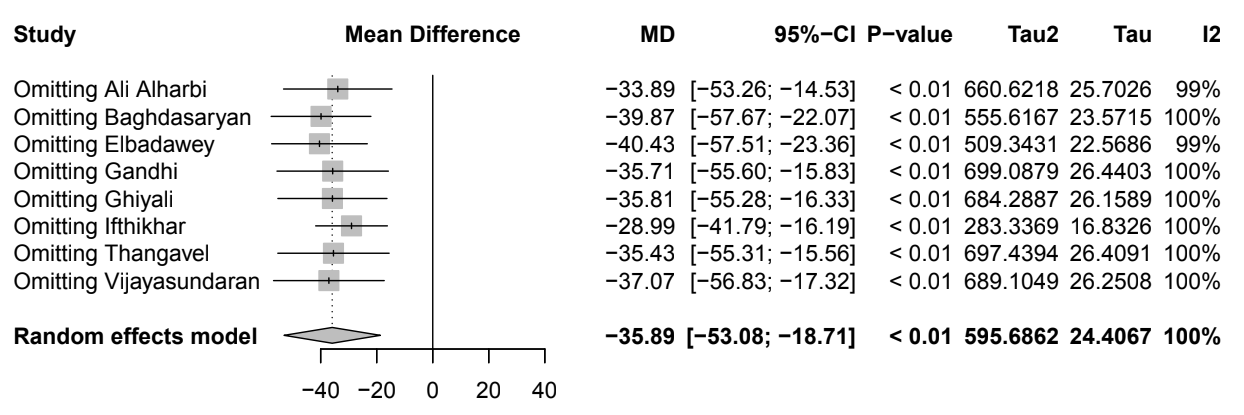


**Figure S8: Leave-one-out analysis for the outcome of intraoperative blood loss.** Each line shows the meta-analysis for this outcome without including the omitted study (according to the first column on the left). Excluding any of the individual studies changed statistical significance or heterogeneity importantly.


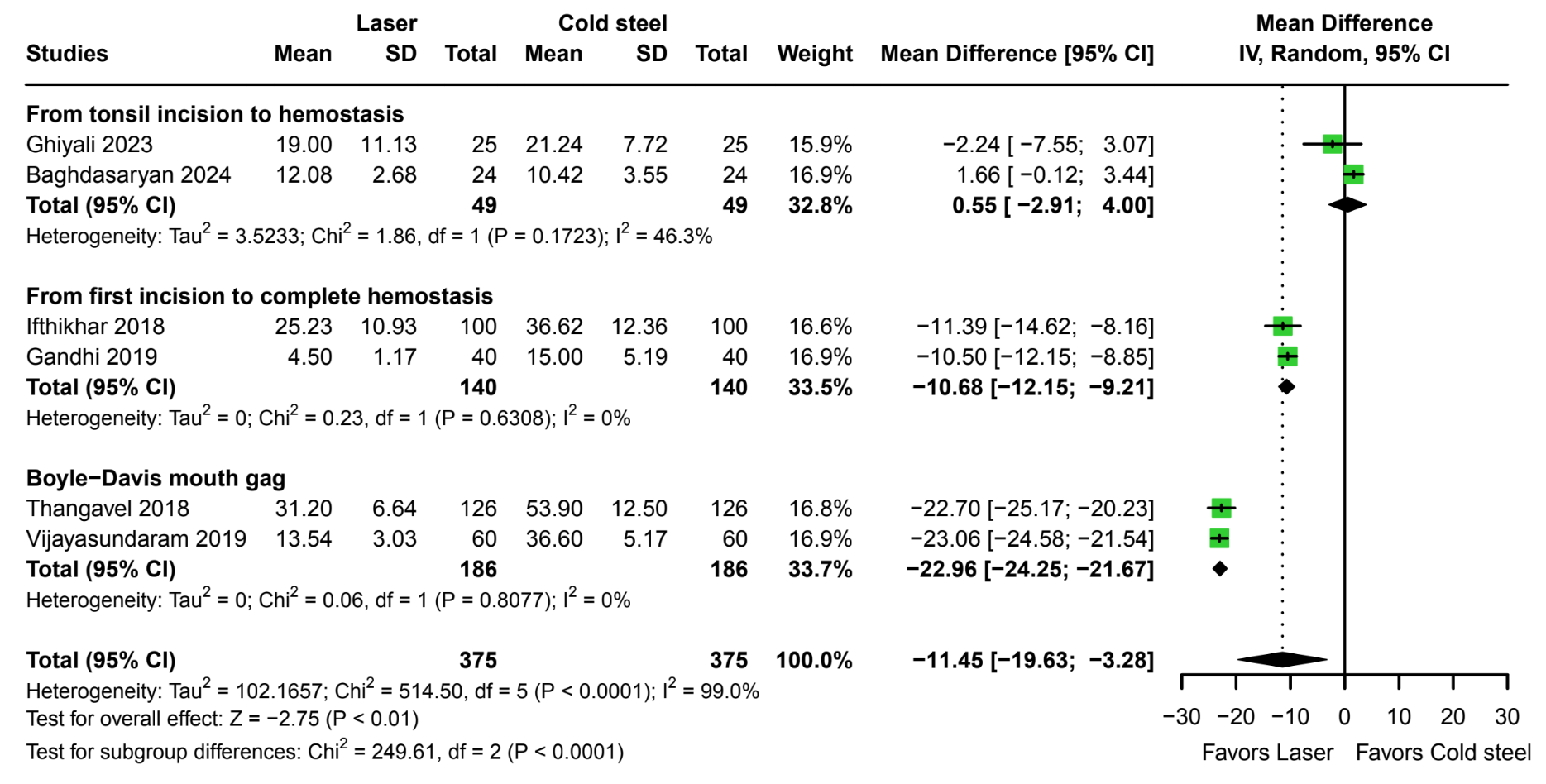


**Figure S9: Subgroup analysis of measurement definitions for the outcome of operation time.** Subgroups are classified by the following criteria: (1) From tonsil incision to hemostasis, in which studies that performed within-subject surgical design are included; (2) From first incision to complete hemostasis, in which studies performing bilateral tonsillectomy with the same technique are included, and considered the time between the first incision of the first tonsil until hemostasis of the second tonsil; (3) Boyle-Davis mouth gag, in which studies performing bilateral tonsillectomy were included, and considered the insertion and removal of the mouth gag to define operation time. On the left side of the figure, each horizontal line represents an individual included study. On the right side of the figure, the dashed vertical line represents the meta-analysis overall measure of effect. The heterogeneity within each subgroup is not significant (I^2^ = 0-46%). Subgroup effect is evidenced with statistically significant subrgoup difference (P < 0.0001). SD: Standard deviation; CI: Confidence interval; IV: Inverse variance.


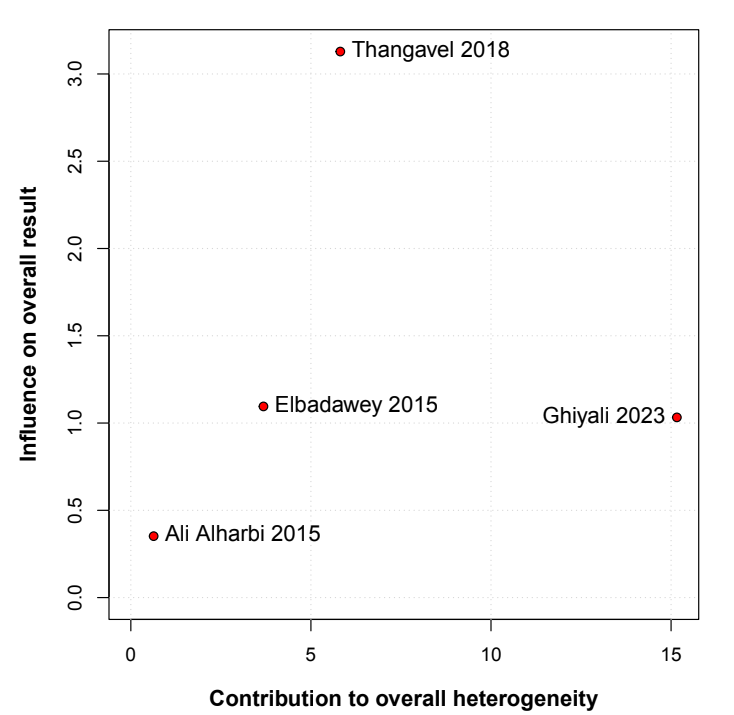


**Figure S10: Baujat plot for the outcome of pain on postoperative day 1.** On the x-axis, the contribution of a study to heterogeneity is presented, while on the y-axis, the influence of this study to the overall heterogeneity. The location of each study on the plot represents how impactful this study is to the overall analysis.


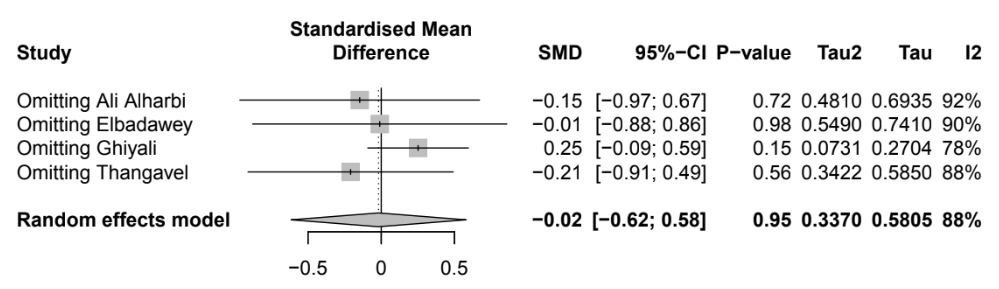


**Figure S11: Leave-one-out analysis for the outcome of pain on postoperative day 1.** Each line shows the meta-analysis for this outcome without including the omitted study (according to the first column on the left). Excluding any of the individual studies changed statistical significance or heterogeneity importantly.


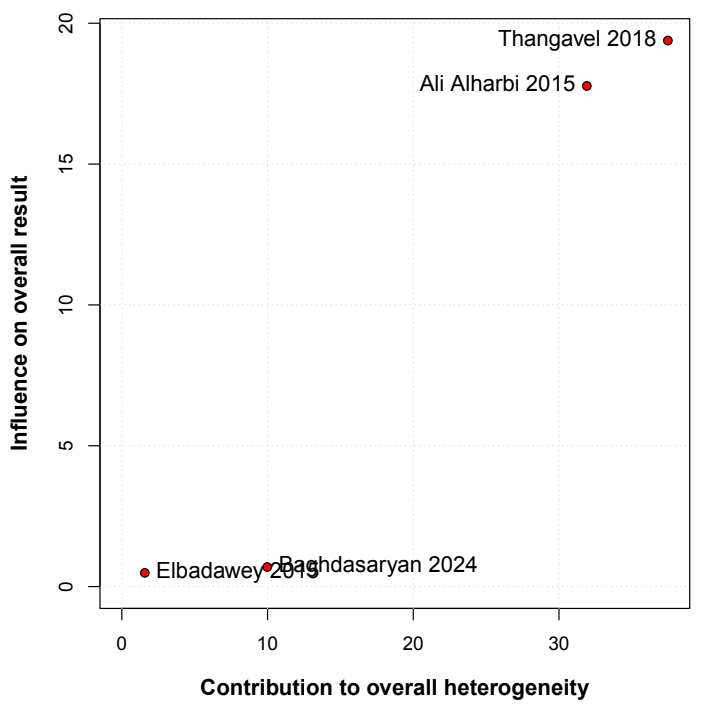


**Figure S12: Baujat plot for the outcome of pain on postoperative day 7.** On the x-axis, the contribution of a study to heterogeneity is presented, while on the y-axis, the influence of this study to the overall heterogeneity. The location of each study on the plot represents how impactful this study is to the overall analysis.


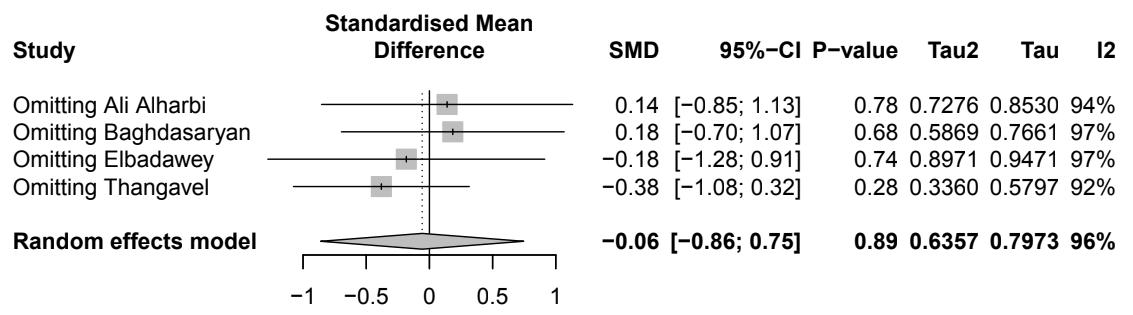


**Figure S13: Leave-one-out analysis for the outcome of pain on postoperative day 7.** Each line shows the meta-analysis for this outcome without including the omitted study (according to the first column on the left). Excluding any of the individual studies changed statistical significance or heterogeneity importantly.


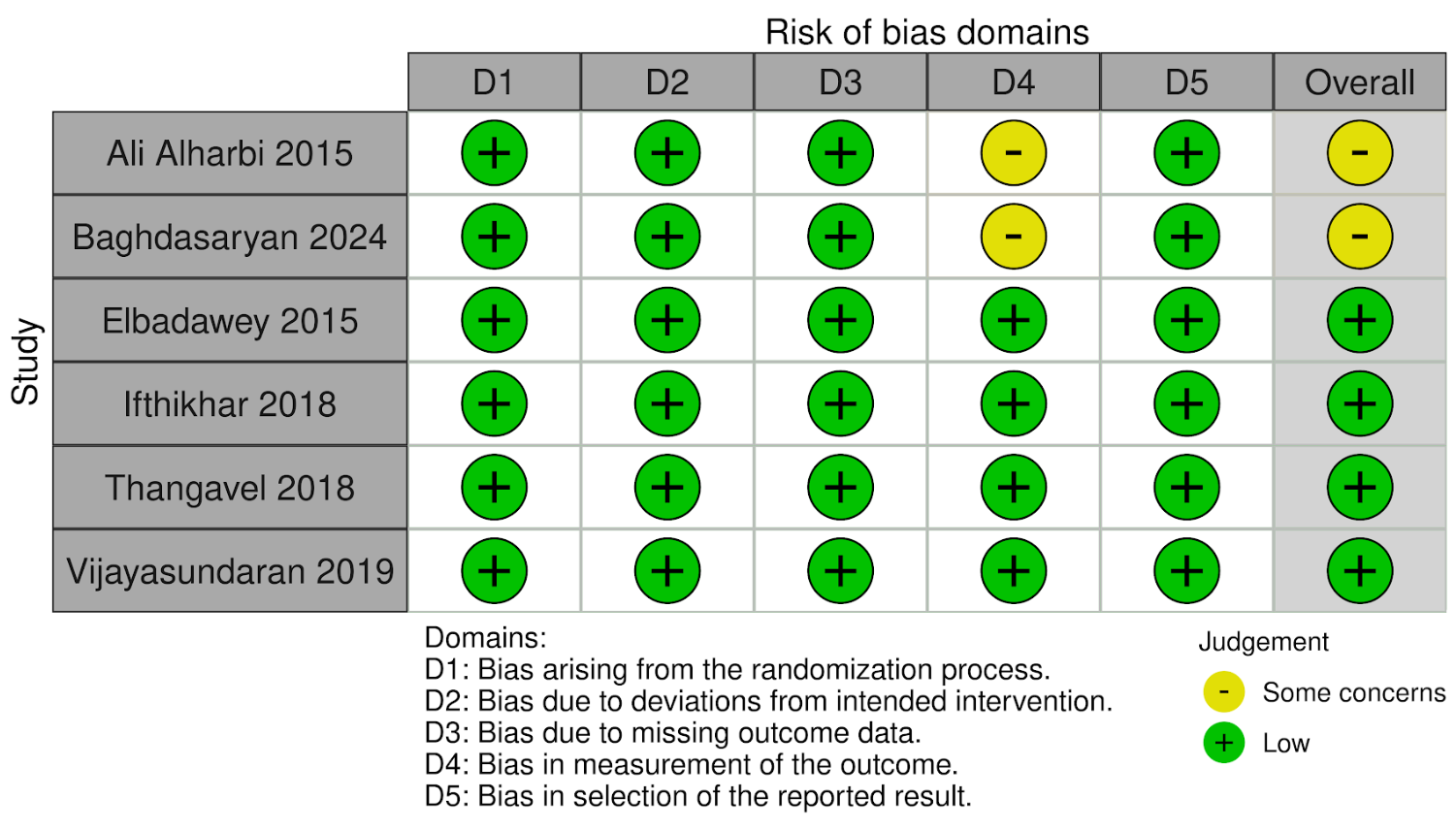


**Figure S14: Risk of bias assessment of randomized controlled trials.**


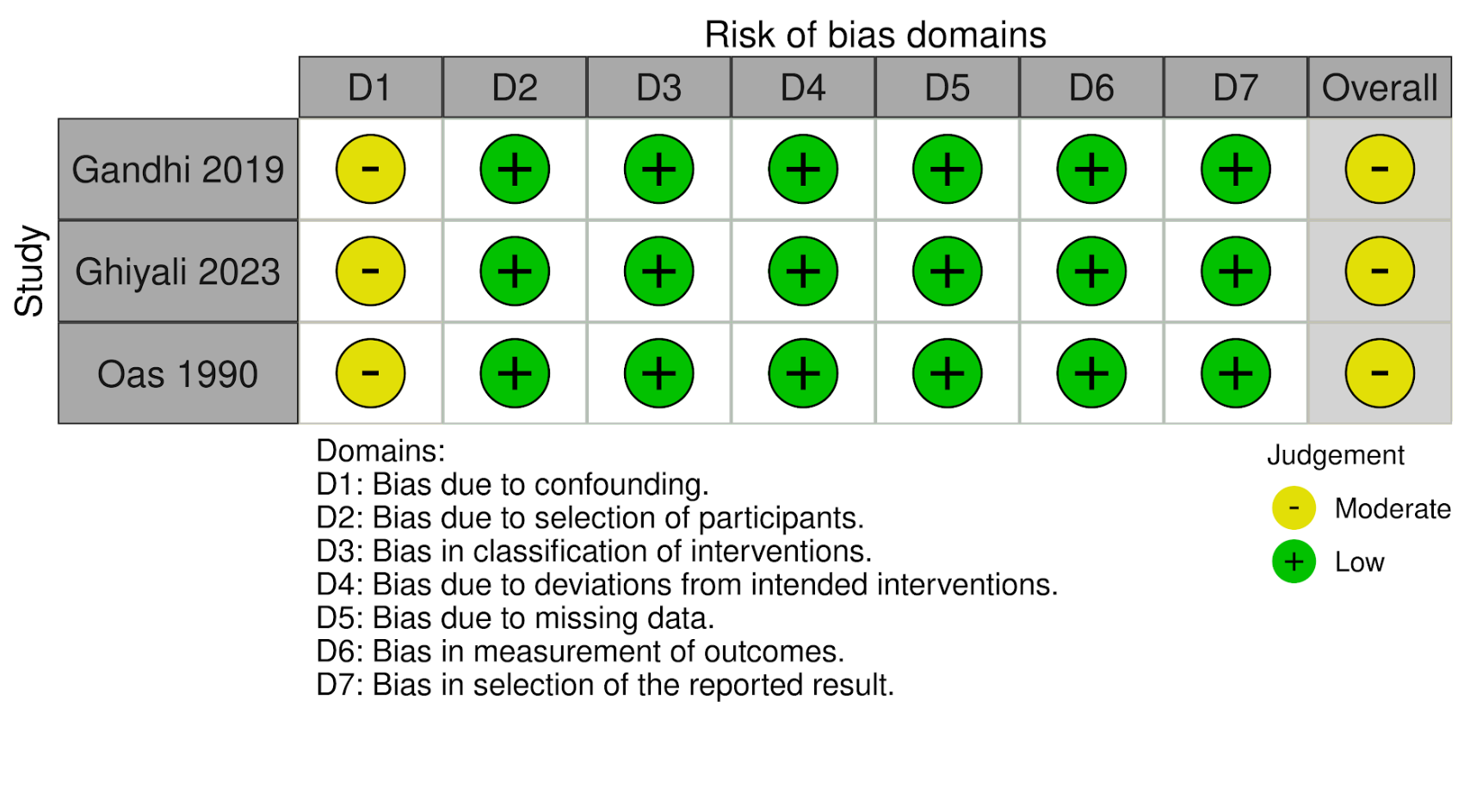


**Figure S15: Risk of bias assessment of non-randomized studies.**


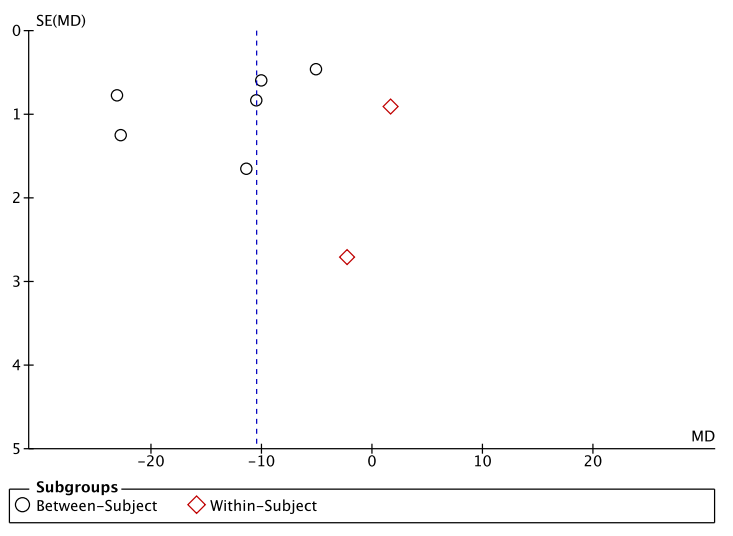


**Figure S16: Funnel plot for the outcome of operation time.** Each study included in the meta-analysis is represented by a circle or square on the scatter plot. The X-axis represents the measure of effect of each study, and the Y-axis represents their corresponding standard error (SE). Visual inspection of this plot shows an asymmetric distribution of studies.

**Supplemental Tables**

**Table S1: Definitions of each study for measuring the outcome of intraoperative blood loss.**

| **Study** | **Measurement method of intraoperative blood loss** |
| --- | --- |
| **Ali Alharbi 2015** | No information |
| **Baghdasaryan 2024** | Suction bottle volume and weight of surgical sponges |
| **Elbadawey 2015** | Weight of saturated swabs and volume of suction bottle |
| **Gandhi 2019** | Every cotton ball fully soaked during surgery represented 5ml of bleeding. They counted the cotton balls and determined approximated volume |
| **Ghiyali 2023** | Weight of saline soaked gauze pieces before and after the surgery, further calculation based on blood density and the volume in suction jar |
| **Ifthikhar 2018** | No information |
| **Oas 1990** | No information |
| **Thangavel 2018** | Weight of cotton balls before and after the surgery, further calculation based on blood density and volume in suction bottle (subtracted the initial saline volume) |
| **Vijayasundaram 2019** | Weighing swabs before and after surgery, and volume in suction bottle |

**Table S2:** **Definitions of each study for measuring the outcome of operation time.**

| **Study** | **Measurement method of operation time** |
| --- | --- |
| **Ali Alharbi 2015** | No information |
| **Baghdasaryan 2024** | Beginning to end of the tonsil removal on each side separately |
| **Elbadawey 2015** | From start of surgery to the removal of the Boyle-Davis mouth gag (includes tonsil removal and hemostasis) |
| **Gandhi 2019** | Total time consumed during surgery (duration of surgery, not further specified) |
| **Ghiyali 2023** | From start of incision to complete hemostasis after tonsil removal |
| **Ifthikhar 2018** | From first incision to the complete hemostasis after tonsil removal |
| **Oas 1990** | No information |
| **Thangavel 2018** | From insertion of the Boyle-Davis mouth gag to the removal of the mouth gag after tonsil removal |
| **Vijayasundaram 2019** | From insertion of the Boyle-Davis mouth gag to the removal of the mouth gag after tonsil removal |
